# Inclusive education in a refugee camp for children with disabilities: How are school setting and children’s behavioral functioning related?

**DOI:** 10.1186/s13031-022-00486-6

**Published:** 2022-10-13

**Authors:** Thomas M. Crea, Elizabeth K. Klein, Oladoyin Okunoren, Maria Paula Jimenez, Greg St. Arnold, Truphena Kirior, Eric Velandria, Daniela Bruni

**Affiliations:** 1 School of Social Work, Boston College, Chestnut Hill, MA USA; 2 Jesuit Refugee Service International Office, Rome, Italy; 3 Jesuit Refugee Service, East Africa Region, Kakuma Programme, Nairobi, Kenya

**Keywords:** Special needs education, Refugees, Refugee camp, Children with disabilities, Educational inclusion, Mainstreaming

## Abstract

Many refugee children face challenges accessing education, but refugee children with disabilities are especially vulnerable to exclusion from school environments as well as social settings. Mainstreaming is considered a best practice but may not always be feasible given the limited resources available in refugee camps. The purpose of this study is to examine the extent to which school setting (i.e., special needs vs. mainstream classrooms) is associated with changes in children’s prosocial behaviors (i.e., social skills and ability to get along well with peers) and behavioral difficulties, accounting for disability status. In Kakuma Refugee Camp in Kenya, researchers collected two waves of data (approximately 2.5 years apart) for students enrolled in special needs schools (*n* = 78) and students who had transitioned from special needs schools into mainstream classrooms (*n* = 51). Children’s average prosocial scores decreased between wave 1 and wave 2, but scores from children in special needs schools decreased at a lower rate indicating potential protective factors in these settings. While children’s average total difficulties decreased over time, children’s difficulties in special needs schools decreased at a faster rate, also indicating potential protective factors. Neither severity of disability nor gender significantly predicted change in prosocial or difficulties scores. In the context of a refugee camp, mainstreaming alone may not fully address the needs of children with disabilities. Specific factors seen in special education settings, such as individualized services, accessible accommodations, and infrastructure supports, must be considered as a means of creating inclusive educational environments.

## Introduction

Of the estimated 83 million forcibly displaced people worldwide, 42% are children [1] and as many as 15–20% are people with disabilities [2, 3]. Children who are refugees are reported to have disabilities in mobility (7%), cognition (5%), vision (1%), but most commonly anxiety (22%) and controlling behaviors (10%) [4]. The vast majority of refugees live in protracted situations and face limited access to employment and education services [5] except those provided by humanitarian agencies. Children with disabilities in refugee camps are especially vulnerable to stigmatization, exclusion, isolation, and violence. These barriers stemming from their disability limit their abilities to access education, essential services, form relationships with their peers, and foster healthy psychosocial well-being [6, 7, 8].

Inclusive education calls for dismantling barriers that prevent marginalized populations from engaging in mainstream education settings [9]. One strategy of inclusive education is to mainstream children with disabilities into general classrooms. This approach, while promising, assumes that the appropriate infrastructure and practices are in place to support children’s learning and socialization [10]. However, this assumption raises questions in the context of a refugee camp that experiences high student-to-teacher ratios and extremely limited resources and infrastructure, and where the evidence base is currently thin [11]. The current study is designed to fill this gap in the literature by examining whether children’s prosocial and difficulties scores change over time depending on the type of their school placement as well as the severity of their disabilities.

## Background

Education is a basic human right [12] and offers significant protection for vulnerable children, including refugee children and children with disabilities [13]. Schools allow for closer supervision, opportunities for safeguarding, and the ability to identify and address a child’s psychological, social, and medical needs [8]. As such, education can help provide a sense of normalcy for children whose lives have been disrupted by violence [14].

For refugee children, education has been shown to offer protection against military conscription, involvement in the sex industry [15], and child marriage [16]. Education can also promote community resilience, empower young people to live fulfilling lives, and educate them about the larger world [13, 17]. Education also increases psychosocial and cognitive protections [5]. For disabled refugee children, education can offer additional benefits such as reducing marginalization because of stigma around disabilities [18].

Yet, schools in refugee camp face a number of challenges, including limited funding, overcrowding, lack of updated learning materials, insufficient teacher training, and poor technology [14]. Children in refugee camps have much lower educational attainment levels than children worldwide. Only 68% of children in refugee camps attend primary school, compared to 91% worldwide. This number drops to only 34% attendance for secondary schools in refugee camps compared to 84% worldwide (UNHCR 2021). In camps, serving the educational needs of children with disabilities is even more complicated given the lack of necessary resources, personnel, and infrastructure in refugee camps [8, 19].

Little guidance currently exists to guide inclusive education efforts for children with disabilities in protracted displacement situations. A prevailing strategy for children with disabilities is to include them in mainstream educational settings [9]. This approach is designed to address barriers children with disabilities face, including barriers of access and barriers of social discrimination. It is supported and emphasized in human rights treaties, such as the UN Convention on the Rights of the Child, the UN Convention on the Rights of Persons with Disabilities, and the UNESCO Salamanca Statement [20, 21]. In the United States, inclusive education for children with disabilities is associated with higher school enrollment and retention [22], as well as increased academic performance for students without disabilities [23]. It is argued that inclusive education provides more educational opportunities for children with disabilities, as well as decreasing discrimination and stigmatization with peers and within the community [24]. A qualitative study in Jordanian refugee camps suggested that children with disabilities in mainstream settings were mostly unable to meet these student’s needs. Mainstream schools suffered from high student–teacher ratios, lack of appropriate facilities, resources, and services,and limited teacher qualifications [25].

Refugee children with disabilities may face discrimination within the home as well. Mothers may experience blame and stigmatization for child’s disability, and one study reported that fathers in one refugee camp commonly would abandon their families if a child was born with a disability [24]. Children may be ostracized by neighbors and extended family, and may be hidden away from community activities [24]. This stigmatization not only isolates children with disabilities in their families and communities, but also limits their ability to access education and opportunities for socialization.

In schools, children with disabilities benefit not only from socialization, but also from specialized services such as individualized case management, targeted outreach, and early childhood interventions [8, 24]. These services are limited in mainstream refugee camp schools, however, because of overcrowded classrooms and low levels of teacher training [19, 26]. Children with disabilities may experience physical difficulties accessing schools, classrooms, and latrines [8]. In addition, children with disabilities frequently experience discrimination from their peers or from their teachers [19]. One study across multiple refugee camps in Africa, Asia, and the Middle East found that children with disabilities in mainstream classrooms had lower attendance and higher dropout rates [8], perhaps related to accessibility challenges or discrimination from peers. A group model building study in Kakuma by our research team [27] found that teachers and parents did not believe greater inclusion directly led to improved children’s well-being. However, these participants welcomed mainstreaming as an opportunity for greater socialization, assuming that proper supports were in place [10, 27]. Another study by our team in Kakuma (*n* = 146; [11] found that children in mainstream schools fared better in their prosocial behaviors (i.e., social skills and ability to get along well with peers) than those in special needs schools, but that this difference disappeared when factoring in the level of children’s emotional and behavioral problems. This study reinforced the need to focus on both inclusion for children with disabilities, as well as initiatives to improve the learning environment, facilities, and infrastructure [11, 28].

For inclusion to happen at the necessary scale, mainstream schools must be welcoming and accommodating towards children with disabilities, efforts that can be difficult when resources are highly limited. Serving the needs of children with varying disabilities requires individualized supports, including (among others): various forms of accommodations within the classroom, communication provided in multiple formats, stigma-reduction campaigns with school personnel and peers, and capacity-building for teachers and staff [29]. Creating inclusive classrooms in takes time and resources, including policy changes, organizational collaboration and teamwork among various stakeholders, teacher training, and sensitization and education for parents [30]. Yet, it can be challenging to implement such changes in refugee camp schools, given the lack of necessary resources, personnel, and infrastructure [8, 19]. Schools designed specifically for students with special needs therefore aim to fill an important gap—addressing immediate needs of education and socialization of children with disabilities in a destigmatized, accommodating space, while larger systemic changes are planned to ensure greater inclusion [28].

### Purpose

The purpose of the current study is to examine whether children’s prosocial behaviors and overall difficulties change over time, based on school setting and disability status, using a longitudinal design. This study builds on the first wave of data collection [11], providing one of the only empirical and longitudinal examinations of the relationship between educational settings and the functioning of children with disabilities who live in refugee camps.

#### Research questions

This study is guided by the following research questions: (1) To what extent does placement in a mainstream classroom influence children’s prosocial behavior score over time, as compared to children in a school specifically for children with disabilities? And (2) To what extent does placement in a mainstream classroom influence children’s total difficulties score over time, as compared to children in a school specifically for children with disabilities?

### Setting

Kakuma Refugee Camp is located in northwestern Kenya in Turkana, which is the poorest county in Kenya [31]. The camp was established in 1992 after the arrival of 12,000 Sudanese “lost boys” who came to Kenya seeking asylum. The camp now hosts refugees from South Sudan, Sudan, Ethiopia, Burundi, the Democratic Republic of Congo, and many others [32]. The camp was designed to host 70,000 people; today, Kakuma and the adjacent Kalobeyei Integrated Settlement have a population of 196,666 registered refugees and asylum seekers [33]. Over half of the refugee population in the camp are children under age 17, with 36% of children under 11 years of age [32].

A majority of Kakuma refugees (68%) and Kalobeyei residents (65%) live below the $1.90/day poverty line. Turkana has an even higher poverty rate, at 72%, compared to the 37% of Kenyans living in poverty at the national level. Most residents near Kakuma live semi-pastoral and nomadic lives, living in huts without amenities such as running water or electricity [34]. Residents of the local Turkana communities surrounding Kakuma may interact with refugees in Kakuma, as well as services available in the camp. These services include some access to educational facilities originally designed for refugees [35] particularly in the nearby Kalobeyei Settlement.

#### Jesuit Refugee Service Special Needs Centers

The JRS Special Needs Centers in Kakuma began in 2010 to provide protective service and nutritional support for people with disabilities. By 2016, they expanded programming to four centers, and expanded their focus as well to include educational and socialization activities. One main objective of the programs was to increase coordination with the formal education system, in order to provide pathways for children to be integrated into these formal systems. To help with these goals, JRS employs a coordinator trained as a special needs educator, increased the specialized training that staff at the Special Needs Centers received, and constructed infrastructure to support these changes and accommodate participants’ special needs. By 2020, JRS opened their fifth center in Kakuma, and served a total of 250 children with disabilities across the centers.

## Methods

### Sample

A baseline survey was conducted in early 2019 in the four JRS Special Needs Centers in Kakuma, with data being collected from parents of children who were currently enrolled in the centers or who had formerly been enrolled in the centers but then transitioned to mainstream classrooms in one of six formal educational centers (*n* = 146). A second wave of data were in September 2021 with additional questions related to disability status and parent engagement. Of the 146 parents sampled during wave 1 of the study, 17 (11.6%) were lost to follow up because of outmigration for a paired sample of *n* = 129. Of these, *n* = 78 attended JRS schools and *n* = 51 attended mainstream schools. The mean age of children was 14.0 (SD = 4.2) and 58.9% were boys; these did not differ to a statistically significant degree between special needs and mainstream schools (see Table 1).

**Table 1:** Covariates by school setting

Variable	Percent or mean (SD)		
	Total (*n* = 129)	JRS school (*n* = 78)	Mainstream school (*n* = 51)
Age**	14.0 (4.2)	13.1 (4.3)	15.4 (3.8)
Gender			
Male	58.9%	55.1%	64.7%
Female	41.1%	44.9%	35.3%
Disability status			
Disability	65.1%	66.7%	62.8%
No disability	34.9%	33.3%	37.3%
Prosocial scores			
Wave 1***	6.4 (2.7)†††	5.4 (2.8)	8.0 (1.9)†††
Wave 2	4.6 (2.6)†††	4.7 (2.6)	4.4 (2.6)†††
Total difficulties			
Wave 1***	20.2 (5.1)†††	21.4 (4.7)†††	18.3 (5.1)†
Wave 2	17.7 (4.4)†††	18.1 (4.8)†††	17.1 (3.6)†

**p < 0.01, ***p < 0.001 between JRS and mainstream

†*p* < 0.05, †††*p* < 0.001 between wave 1 and wave 2

### Procedures

Data were collected by trained local research assistants in English, Swahili, or Arabic, depending on the native language of the participant. All participants provided informed consent prior to their participation and were given a small cash incentive upon completion of the survey. All study protocols were approved by the [university] Institutional Review Board (IRB), the Ethics Review Board of Daystar University, and by the Government of Kenya National Commission for Science, Technology and Innovation (NACOSTI).

In February 2019 (Wave 1), data were collected using paper and pencil, and survey data were later entered into an electronic database. In September 2022 (Wave 2), data were collected using electronic tablets on the REDCap platform. Local research assistants were trained on the protocol, instruments, platform, and the devices used. Each data collection effort occurred over a two-week period.

### Measures

#### Dependent variables

There are two dependent variables used in this study, both taken from the child’s “Parent Completed Strengths & Difficulties Questionnaire (SDQ)” [36]. The SDQ is a behavioral screening measure to assess the following domains: (1) emotional symptoms (5 items); (2) conduct problems (5 items); (3) hyperactivity/inattention (5 items); (4) peer relationship problems (5 items); and (5) prosocial behavior (5 items).

The Prosocial Scale comprises five items on a 3-point Likert scale (0 = Not True, 1 = Somewhat True, 2 = Certainly True) for a composite score ranging from 0 to 10. Internal consistency in this sample for Prosocial Scores was good with α = 0.72. Prosocial behaviors include items such as, “Considerate of other people’s feelings” and “Shares readily with other children,” that measure the child’s ability to get along well with other children.

The Total Difficulties Scale comprises all the questions in the survey excluding the prosocial scores: emotional symptoms, conduct problems, hyperactivity/inattention, and peer relationship problems. The 20 questions that make up the Total Difficulties Scale are also on a 3-point Likert scale (0 = Not True, 1 = Somewhat True, 2 = Certainly True). Internal consistency in this sample for Total Difficulties was moderate with α = 0.59.

#### Independent variables

The independent variables are child's gender (0 = male, 1 = female), disability status (0 = no disability, 1 = disability), age (in years), type of school setting (0 = JRS, 1 = mainstream school), and time (0 = Wave 1, 1 = Wave 2). Disability data was determined based on the Washington Group Short Set on Functioning (WG-SS) [37], which asked questions determining the degree of difficulty with Vision, Hearing, Mobility, Concentration, Self-Care, and Communication. Each question was scored using a 4-point Likert scale: 1 = no difficulty, 2 = some difficulty, 3 = a lot of difficulty, and 4 = cannot do at all. According to Washington Group scoring instructions [37], cases were coded as “disabled” if any of the individual items was scored a 3 (a lot of difficulty) or 4 (cannot do at all).

### Analysis

All analyses were completed by using Stata 15. Bivariate tests were conducted to examine differences between variables (see Table 1). Two Stepwise OLS regression analyses were completed to assess the relationship of the covariates (gender, disability status, age, type of school setting, and time) and the dependent variables of Prosocial Behaviors (Model 1), and Total Difficulties (Model 2), respectively. An interaction effect between time (Wave 1 × Wave 2) and the school setting was included in both models, to see how school setting may have affected the scores in change over time. Robust standard errors were applied to account for clustering between time points within cases. Using Stata syntax *marginsplot*, we plotted the interaction between time and school for prosocial scores and for total difficulties scores.

## Results

Descriptive statistics are presented in Table 1. 65.1% were categorized as having a high level of disability and this status did not differ between school type to a statistically significant degree. Mainstream students were two years older on average than JRS students (*p* < 0.01). Prosocial Behavior scores were higher for mainstream students compared to JRS students during Wave 1 (*p* < 0.001). Prosocial Behaviors also decreased between Wave 1 and Wave for the overall sample (*p* < 0.001) and for mainstream students (*p* < 0.001); these also decreased for JRS students but not to a statistically significant extent. Total Difficulties scores were higher for JRS students than mainstream students in Wave 1 (*p* < 0.001). Total Difficulties also decreased between Wave 1 for the overall sample (*p* < 0.001), for JRS students (*p* < 0.001), and for mainstream students (*p* < 0.05).

Descriptive statistics for disability type are presented in Table 2. The most prevalent disability was Cognitive for all students (45%), for JRS students (49%), and for mainstream students (41%), respectively. The next most prevalent disability for JRS students was self-care disability (33%) and communication disability (32%). For mainstream students, the second-most prevalent disability was mobility disability (18%) followed by self-care disability (16%). JRS students had, on average, 1.5 disabilities (*SD* = 0.2) and mainstream students had on average 1.0 disability (*SD* = 0.2). Students in JRS classrooms had higher levels of disability in Communication (*p* < 0.05) and Self-Care (*p* < 0.05) than students in mainstream classrooms.

**Table 2:** Disability types by school setting

Variable	Total (*n* = 129) (%)	JRS (*n* = 78) (%)	Mainstream (*n* = 51) (%)
Vision			
Not visually disabled	95	95	96
Visually disabled	5	5	4
Hearing			
No hearing disability	91	94	88
Hearing disability	9	6	12
Mobility			
No mobility disability	78	74	82
Mobility disability	22	26	18
Communication*			
No communication disability	75	68	86
Communication disability	25	32	14
Self-care*			
No self-care disability	74	67	84
Self-care disability	26	33	16
Cognition			
No cognitive disability	55	51	59
Cognitive disability	45	49	41
Overall disabilities [M (SD)]	1.3 (0.1)	1.5 (0.2)	1.0 (0.2)

*p < .05

**Fig. 1:**
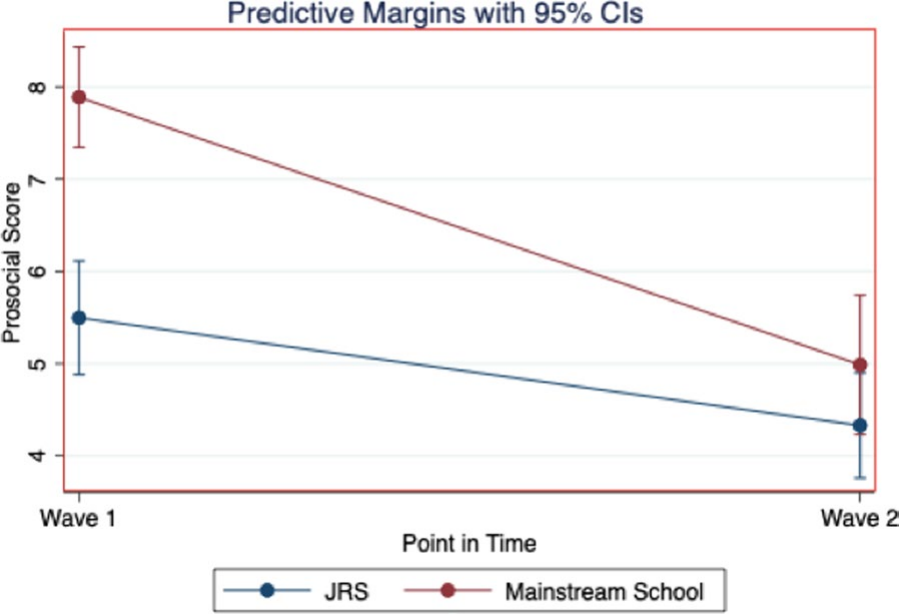
Change in prosocial scores by school type

Results for Model 1 (see Table 3) showed that Prosocial Behaviors varied significantly between schools, with students at mainstream schools predicted to report higher Prosocial Behaviors than students at JRS schools (*p* < 0.001). Age, gender, or disability status were not significantly associated with Prosocial Behaviors. Change in Prosocial Behaviors over time was also a function of which type of school a student attends (*p* < 0.01). Mainstream students started with higher scores at Wave 1, and while both groups decreased between Wave 1 and Wave 2, mainstream students decreased at a faster rate than JRS students (see Fig. 1).

**Table 3:** Regression tables: interaction between time and school

Predictor	Prosocial (*n* = 129)		Total difficulties (*n* = 129)	
	*b* (SE)	95% CI	b (SE)	95% CI
Time (compared to Wave 1)				
Wave 2	− 0.17 (0.41)**	[− 1.96, 0.37]	− 3.43 (0.78)***	[− 4.91, − 1.90]
School (compared to JRS)				
Mainstream	2.39 (0.42)***	[1.55, 3.23]	− 2.60 (0.86)**	[− 4.30, − 0.90]
Time # school	− 1.73 (0.62)**	[− 2.95, − 0.52]	2.37 (1.08)*	[− 0.23, 4.50]
Disability	− 0.08 (0.33)	[− 0.74, 0.58]	− 0.49 (0.65)	[− 0.80, 1.78]
Gender	0.18 (0.33)	[− 0.48, 0.84]	0.24 (0.62)	[0.00, 1.47]
Age (years)	0.07 (0.04)	[− 0.01, 0.15]	− 0.19 (0.07)**	[− 0.33, − 0.06]
Constant	4.49 (0.66)***	[3.21, 5.78]	23.48 (1.16)***	[21.18, 25.78]

*p < .05, **p < .01, ***p < .001

**Fig. 2:**
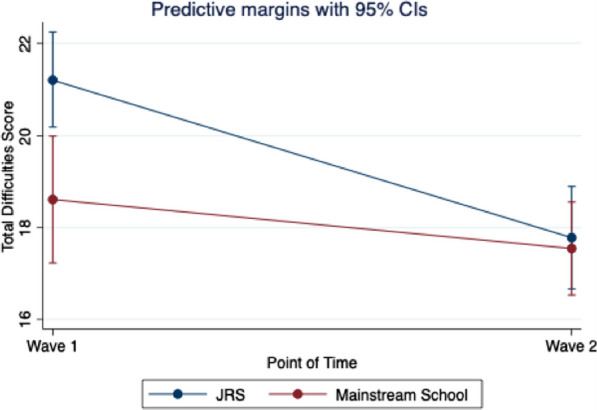
Change in total difficulties scores by school type

Results for Model 2 (see Table 3) showed that Total Difficulties varied significantly between schools, with students at mainstream schools predicted to report lower Total Difficulties than students at JRS schools (*p* < 0.01). Higher age was associated with lower Total Difficulties (*p* < 0.01) but there was no association between disability status, or gender, and Total Difficulties. Change in Total Difficulties over time was also a function of which type of school a student attends (*p* < 0.05). JRS students started with higher Total Difficulties scores at Wave 1, and while both groups decreased between Wave 1 and Wave 2, JRS students decreased at a faster rate than mainstream students (see Fig. 2).

## Discussion

In refugee camp settings, children’s access to education can buffer the adverse effects of displacement and resettlement by creating a sense of normalcy, reducing their exposure to risky situations, and aid in the acquisition of knowledge [38]. For refugee children with disabilities, higher levels of stigmatization and social exclusion stand as significant barriers to education access and participation. Yet, inclusion for children in these contexts may not necessarily equate to mainstreaming, absent the proper supports [28]. Rather, inclusive practices should also include lowering stigmatization and increasing opportunities for socialization, even when mainstreaming opportunities are not currently viable [8, 27].

This study found that children in special needs classrooms showed greater difficulties and lower prosocial scores than children in mainstream classrooms, similar to findings from our earlier baseline study [11]. This finding suggests that special needs classrooms serve children with higher levels of need. Yet, the difference in prosocial behaviors decreased over time, such that by Wave 2, children in special needs classrooms were statistically equivalent to children in mainstream classrooms. A similar dynamic was discovered for children’s total difficulties over time. Children in mainstream schools reported lower total difficulties scores at Wave 1, as might be expected. By Wave 2, children’s scores decreased more rapidly in special needs schools than in mainstream schools, with students at the two schools having nearly equal scores by Wave 2.

There may be several explanations for these changes over time. First, Wave 1 data were collected prior to the COVID-19 pandemic, while Wave 2 data were collected 1.5 years into the pandemic. We surmise that the overall decrease in prosocial scores could be related to the pandemic, which has been noted to affect children's education and well-being negatively [39, 40]. In JRS schools, the pandemic necessitated temporary school closures, home-based visits by teachers, restricted class sizes, and outdoor teaching when in school. Assuming this hypothesis is correct, the environment of special needs classrooms may have buffered the negative effects of the pandemic.

Prosocial behaviors for children in special needs classrooms declined significantly less steeply than those in mainstream classrooms. We believe that the reasons for these buffering effects are as follows: (a) special needs classrooms have a significantly smaller number of students per class and a lower teacher-student ratio; (b) all children in special needs classrooms experience some form of disability, which may help them feel less marginalized; and (c) JRS schools in Kakuma are often the first point entry to the school environment for children with disabilities, such that they are “eased” into the school environment before moving towards mainstream schools. Further research is needed, however, to examine the most effective components of special needs classrooms in promoting children’s well-being, and to see if these can also be implemented in mainstream settings, if they are not already.

The steeper decrease in difficulties for children in special needs schools suggests that these classrooms are providing a beneficial environment for children. Importantly, this difference is not driven by greater resources or funding. JRS schools receive less funding than mainstream schools, because up until recently they were classified as non-formal settings. Yet, classrooms in JRS schools contain fewer students so that teachers are able to approach instruction from a more individualized perspective. Such an approach is impossible in schools with large student–teacher ratios. JRS teachers also receive training and supports (or “formation”) to increase their skills but also to instill values of accompaniment and humanity in their approach to children with special needs. This finding raises important questions about how we define inclusion for children with disabilities [27], and whether mainstreaming alone is a sufficient approach to ensuring children’s well-being [10]. It is possible that in a highly resource constrained context like Kakuma, special needs schools are better equipped to serve children with higher levels of need because teachers can focus specifically on the needs of children with disabilities. In addition, smaller classrooms, a chance at socialization, peer and adult acceptance, and possible lower levels of stigmatization, all may be driving factors in the benefits of education in special needs classrooms.

In spite of the apparent protective factors in special needs classrooms, we are not arguing *against* mainstreaming children with disabilities per se. Even with a relative lack of empirical evidence, mainstreaming is in keeping with Article 24 of the United Nations Convention on the Rights of Persons with Disabilities [21] that promotes inclusive education at all levels. The findings of our study suggest that we focus on improving the infrastructure in refugee camps to support a safe and inclusive learning environment for children with disabilities. In contexts such as Kakuma where schools are typically under resourced [19, 26], it may make sense to develop a graduated system, where children are first educated in more contained settings before “graduating” to mainstream classrooms. That system may already be implicit in Kakuma but more needs to be done to operationalize and implement this system. For example, JRS offers two categories of education supports: a specialized curriculum (for children who will likely not be able to manage in a mainstream setting given cognitive and related disabilities) and a preparation curriculum that offers support for children who eventually will likely transition to a mainstream setting. This type of customized support may serve as the basis for a more well-defined pipeline between special needs and mainstream schools.

In any classroom serving children with special needs, however, teachers need to be trained in specialized skills, classrooms should be physically accessible, and issues around stigmatization and acceptance need to be proactively addressed. These changes to infrastructure will be necessary in mainstream classrooms if children with disabilities are to be integrated into such settings. One approach that focuses on changing infrastructures and systems is twin-tracking [11, 28]. Twin-tracking is as a concurrent method of incorporating (a) disability-specific and oriented and (b) disability-inclusive initiatives to address the rights of people with disabilities and to ensure that education initiatives are inclusive and are informed by those with and without disabilities [28]. Such an approach focuses on meeting the needs of individual students, while also addressing the systemic barriers that prevent students from full inclusion.

Other findings from this study bear mentioning. School setting proved to be a more powerful predictor of children’s well-being than the level of children’s disability. Disability status did not emerge as a significant predictor of either prosocial behaviors or total difficulties, nor did disability status interact with time to influence these variables. Disability status also did not differ between special needs and mainstream schools to a statistically significant degree. Also, no differences emerged based on children’s gender. This is an encouraging finding given the noted gender disparities in education based on refugees’ countries of origin in Kakuma [17]. More research should be done, however, to examine gender representation in schools more broadly in Kakuma, as selection bias likely exists on which girls are able to attend school.

### Limitations

This study has limitations. This longitudinal study occurred before and during the COVID-19 pandemic, so it is difficult to isolate the effects of the pandemic from our variables of interest. There was a significant amount of attrition from Wave 1 to Wave 2 mostly because of outmigration. Data collection during Wave 1 was paper-based and administered by research assistants with the principal investigator providing on-site supervision. Wave 2 data were collected electronically with remote supervision by the research team, given pandemic travel restrictions. This difference may have affected the study’s findings in unknown ways. This study’s findings may not be generalizable to all refugee camp contexts given the unique nature and populations of each camp.

## Conclusion

This study’s findings suggest that the more contained environments offered by special needs classrooms may provide some benefit to children with disabilities in a refugee camp. A graduated approach to preparing children with disabilities may ensure that children’s gains are not lost if and when they transition to mainstream classrooms. A twin tracking approach [11, 28] is needed to improve infrastructure in refugee camp classrooms, to ensure that the needs of children with disabilities are adequately met. Future research should examine each the dynamics of school settings in more detail, to identify effective strategies to inclusive education for children with disabilities. Future research should also include examinations of parental involvement for a fuller understanding of how children with disabilities function in school settings, given links between parental involvement and greater emotional wellbeing of children, parent-teacher relationships, achievement, and academic outcomes [41, 42, 43, 44]. Such home-school partnerships could help diminish the stigma of disabilities in this context and help ensure a more seamless transition for children to the most appropriate school setting.

## Data Availability

Data are not publicly available given the vulnerable nature of the population and the risk of deductive disclosure.
